# Breakpoint Associated with a novel 2.3 Mb deletion in the VCFS region of 22q11 and the role of *Alu *(SINE) in recurring microdeletions

**DOI:** 10.1186/1471-2350-7-18

**Published:** 2006-03-02

**Authors:** Raihan K Uddin, Yang Zhang, Victoria Mok Siu, Yao-Shan Fan, Richard L O'Reilly, Jay Rao, Shiva M Singh

**Affiliations:** 1 Department of Biology, University of Western Ontario, London, Ontario, N6A 5B7, Canada; 2 Division of Medical Genetics, University of Western Ontario, London, Ontario, N6A 5B7, Canada; 3 Department of Psychiatry, University of Western Ontario, London, Ontario, N6A 5B7, Canada

## Abstract

**Background:**

Chromosome 22q11.2 region is highly susceptible to rearrangement, specifically deletions that give rise to a variety of genomic disorders including velocardiofacial or DiGeorge syndrome. Individuals with this 22q11 microdeletion syndrome are at a greatly increased risk to develop schizophrenia.

**Methods:**

Genotype analysis was carried out on the DNA from a patient with the 22q11 microdeletion using genetic markers and custom primer sets to define the deletion. Bioinformatic analysis was performed for molecular characterization of the deletion breakpoint sequences in this patient.

**Results:**

This 22q11 deletion patient was established to have a novel 2.3 Mb deletion with a proximal breakpoint located between genetic markers RH48663 and RH48348 and a distal breakpoint between markers D22S1138 and SHGC-145314. Molecular characterization of the sequences at the breakpoints revealed a 270 bp shared sequence of the breakpoint regions (SSBR) common to both ends that share >90% sequence similarity to each other and also to short interspersed nuclear elements/Alu elements.

**Conclusion:**

This Alu sequence like SSBR is commonly in the proximity of all known deletion breakpoints of 22q11 region and also in the low copy repeat regions (LCRs). This sequence may represent a preferred sequence in the breakpoint regions or LCRs for intra-chromosomal homologous recombination mechanisms resulting in common 22q11 deletion.

## Background

The 22q11.2 region is a hotspot for rearrangements due to deletions, duplications and translocations. These rearrangements result in altered gene dosage [1] and lead to congenital malformations including DiGeorge (DGS; MIM 188400)[2], velocardiofacial (VCFS; MIM 192430)[3], der(22) [4] and cat-eye (MIM 115470) syndromes [5]. The most common of these 22q11.2 microdeletion syndromes is the VCFS/DGS, which occurs with an estimated frequency of 1 in every 4000 live births [6]. It represents a variety of clinical manifestations including learning disabilities, characteristics facial appearance, velopharyngeal insufficiency, hypernasal speech, cleft palate and conotruncal heart defects [3]. A subset of severely affected patients also have a hypoplastic or absent thymus gland and hypoparathyroidism with hypocalcaemia [2]. Most clinical features associated with this disorder show variable expressivity and reduced penetrance [1], however adults with this syndrome commonly develop major psychiatric illnesses, particularly schizophrenia and bipolar disorder [7-11]. The vast majority of patients share a common 3 Mb hemizygous deletion. Except for a few rare cases, the remaining patients have smaller deletions nested within the 3 Mb typically deleted region (TDR) [12].

The molecular nature of the rearrangements responsible for 22q11 microdeletions are related to the genomic structure of the 22q11.2 region, which contains long stretches of repeated sequences clustered together, known as low-copy repeats (LCRs) of >95% identity [13,14]. These chromosome 22-specific LCRs have been reported at or near the breakpoints of the 3 Mb TDR affecting the DGS/VCFS deletion on 22q11.2 [15-20]. They are known to mediate unequal non-allelic homologous recombination events and contribute to the rearrangements associated with genomic disorders [21-23]. Interestingly, not all LCRs of 22q11 appear equally effective in causing microdeletions and they do differ with respect to some of their sequences. Further, LCRs contain highly repetitive elements such as short interspersed nuclear elements (SINEs) and long interspersed nuclear elements (LINEs). These elements, particularly SINEs have been implicated in chromosome rearrangements and disease [24]. Alu elements, part of the SINE family of transposable elements [24], have also been established as having a role in modulating the architecture of the human genome in association with human disorders and in mediating gene rearrangements has been established [25,26]. It is therefore important to assess the nature of individual LCRs associated Alu elements in novel microdeletions.

In this paper we report the result from a case study where we have characterized the deletion region in a VCFS patient. We have identified a novel 2.3 Mb deletion in chromosome 22q11.2 region in the patient. We have also analyzed the sequences at the two breakpoints for possible Alu like elements and identified a shared sequence of the breakpoint regions (SSBR) that may predispose this region to microdeletions.

## Methods

### Patient selection

A patient with a 22q11 microdeletion was identified and gave informed consent to participate in research aimed at increasing knowledge about the deletion.

### Chromosome 22q11 microdeletion analysis

Genomic DNA was extracted from fresh peripheral blood of the patient using QIAamp DNA maxi kit (QIAgen inc.) following the manufacturer's instructions. Genotype analysis within the 22q11 region was conducted to precisely define the chromosome breakpoints. The DNA sequence from this region was used to independently confirm the hemizygousity observed during FISH analysis. It was also used to establish the exact region involved in the patient specific deletion through dosage analysis of markers within the 22q11 region using a total of 40 PCR primer sets [see Additional file 1]. Eighteen of the primer sets represented established sequence-tag site (STS) markers [27] that reside in the 3 Mb TDR. The remaining primer sets were designed within STS markers found to encompass novel proximal and distal breakpoint regions (this study). The patient's marker specific dosage was compared to a matched control. PCR products were analyzed on a 6% polyacrylamide gel to confirm expected PCR amplification. PCR fragments of interest were then isolated and purified from the gel and the identity of the fragment was confirmed by diagnostic restriction enzymes or sequencing. The PCR conditions for all sets of experiments (P) were optimized and contained an internal control (N). PCR product quantitation was carried out in the log phase at three PCR cycles (usually between 20 to 30 cycles) using a gel documentation system (Molecular Analyst 1.5, BioRad Laboratories). A 1N:1P ratio indicated no deletion and a ratio of 2N:1P indicated a marker deletion in the patient. All dosage estimates were based on three independent PCR reactions. This analysis enabled us to narrow down the deletion breakpoint regions while PCR amplification of the recombined DNA and its sequencing allowed us to establish the sequence of the patient specific deleted region.

### Analysis and characterization of the 22q11 breakpoints

The sequencing of the breakpoints allowed us to identify the distal and proximal short sequences involved in any break and re-joining. The genomic sequence of the 22q11 typically deleted region (22qTDR) from genetic marker D22S427 to D22S308 was obtained from the Ensembl human genome database [28]. Repeat sequences were masked using the RepeatMasker tool [29]. Blastn [30] analysis of the sequences at the proximal and distal breakpoints established a shared sequence between the breakpoint regions (SSBR). This SSBR sequence was compared with both the original and repeat masked 22qTDR sequence using BLAST analysis where gapped alignment was applied with low complexity filter on the query sequence. The identity cut off for all comparisons were set to 90% with a minimum length of 200.

Data regarding location, class type, name and any other pertinent information was also obtained for the different repeat types present in the 22qTDR sequence using the RepeatMasker tool. Using custom perl scripts and blast analysis, the data was analyzed to extract the location and name of each type of repeat present in the 22qTDR sequence and to compare the relationship of each repeat to the observed SSBR.

Further analysis involved identification of any association between the SSBR sequence and the sequences around the boundary of the published deleted regions (PDR) (Table 1). For this, a number of custom tracks were developed in .bed file format to individually load the following information in the IGB (Integrated Genome Browser) [31]: 1. LCRs located in the 22qTDR based on the chromosome 22 LCR positions as described in [32]; 2. Locations of SSBRs along the 22qTDR; 3. Locations of different repeat elements; 4. Approximate boundaries of the PDR as estimated from the locations of the genetic markers. These tracks were loaded into the IGB along with the annotation for chromosome 22. This identified a number of occurrences of SSBR sequence within each 50 kb block of 250–300 kb region on both the 5' and 3' ends of the approximate breakpoint position of the PDR.

**Table 1:** Approximate breakpoint positions (based on the known/reported marker positions) of previously established deleted regions on 22q11 region.

ID	Proximal Breakpoint	Distal Breakpoint	Reference
BM-41/308/293	17063468	20207821	Carlson et al 1997
BM-14	17063468	18725000	Carlson et al 1997
BM-8	17383947	18725000	Carlson et al 1997
G	17579237	19600433	Carlson et al 1997
2.3 Mb Deletion	17001842	19356929	Current Study

Data analysis was facilitated by the use of three different custom perl script parsers (available upon request): a) bl2seq_parser.pl: to parse the output of the blast alignment tool; b) rmasked_to_bed.pl: to parse the output of the RepeatMasker tool to extract the location and identity of repeat elements and to convert the results in .bed file format so that they can be loaded to UCSC [33] or IGB genome browser; c) BedAnalyzer.pl: to parse repeat element data file for possible occurrence of SSBR sequence in different repeat families.

## Results

### Clinical characteristics of the patient

This male patient was born to non-consanguineous parents at normal gestation weighing 6 pounds. Pregnancy was uncomplicated with no exposure to known teratogens and delivery was by caesarean section. In early infancy, the patient suffered frequent nasopharyngeal reflux, and speech delay was evident by 3 years of age. Velopharyngeal insufficiency and a submucous cleft palate were identified by nasal endoscopy and as a result the patient underwent pharyngoplasty. Carotid arteries were noted to be medially deviated, however a 2D echocardiogram showed no evidence of a congenital heart defect. His serum calcium levels have been borderline low. At age 18, this individual showed minimal facial expression, had a head circumference at 90^th ^percentile, a weight greater than 98^th ^percentile and a height at 75^th ^percentile. Palpebral fissures were normal in length measuring 3.0 cm (90^th ^percentile). Palate was abnormal with the pharyngeal flap evident. The patient had limited flexion of the thumbs, and deep tendon reflexes were very brisk with unsustained ankle clonus. Academically, he completed a basic level secondary school education and socially tended to be introverted. His mother's chromosome results using the 22q FISH probe were normal. The patient's father was not available for testing.

### Deletion analysis

**Figure 1:**
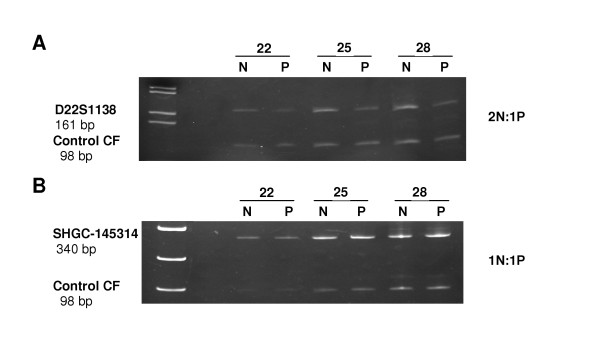
Semi-quantitative PCR analysis of the markers at the proximal and distal breakpoint region of 22q11. Genetic markers D22S1138 (**A**) and SHGC-145314 (**B**) were used against control marker CF (cystic fibrosis). The result showed a control-to-patient intensity ratio of 2:1 and 1:1 respectively. N means normal control and P means patient.

**Figure 2:**
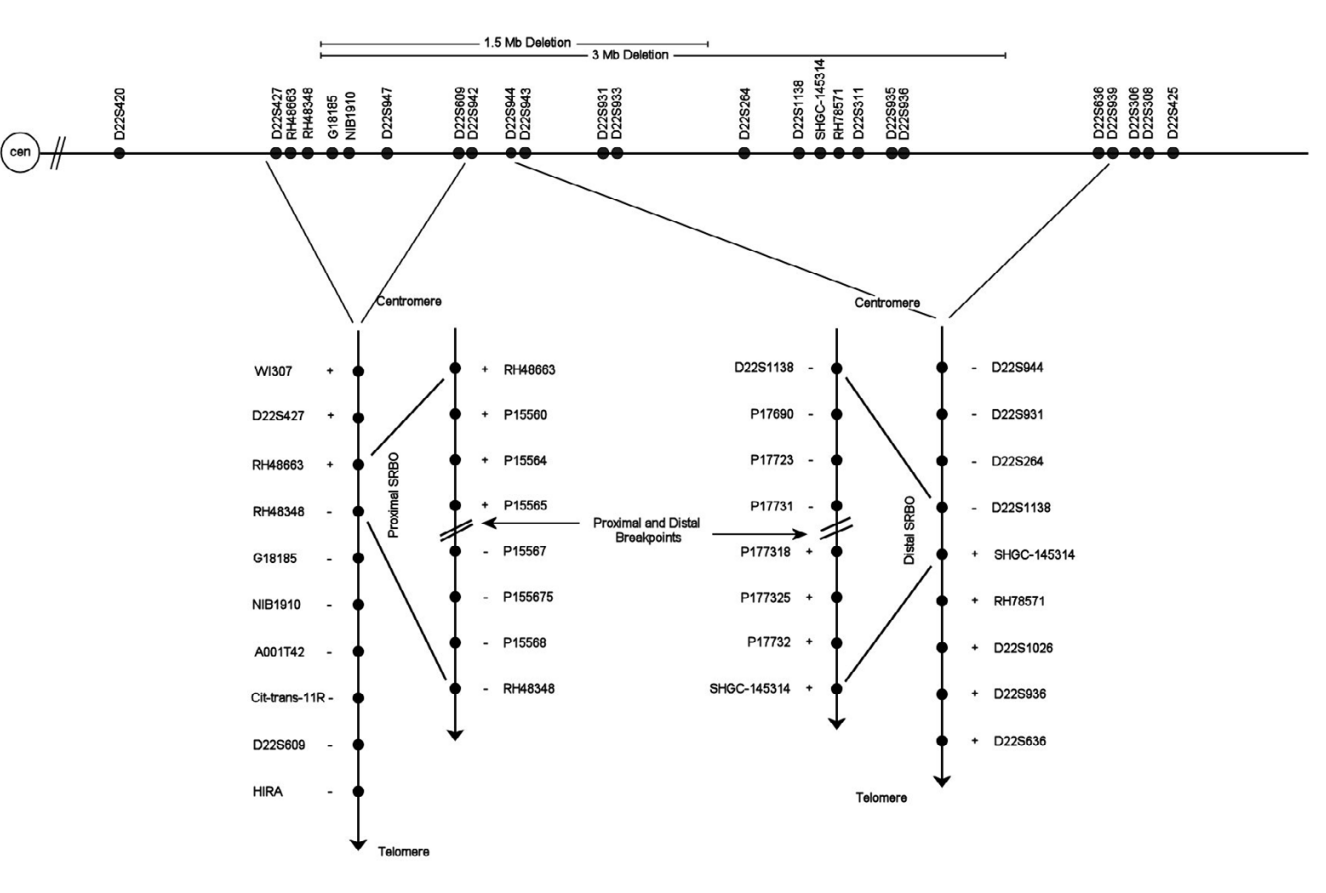
Genetic markers (filled circle) along the chromosome 22q11 region. The 3 Mb and 1.5 Mb typical deletions are marked above the chromosome. Below it are the approximate positions of known genetic and custom primers that were used to interrogate the current novel deletion. Proximal and distal SRBOs, along with the approximate breakpoint positions are also shown.

We performed molecular characterization of the deletion in order to precisely define the interval encompassing the proximal and distal breakpoints responsible for the abnormality. The experimental approach relied on quantitative PCR using a set of 40 unique primers [see Additional file 1] to interrogate the 22q11 region at various intervals starting from the marker WI307 at the proximal end to D22S936 at the distal end. Following amplification, the PCR products were run on a 6% polyacrylamide gel and specific fragments were quantified. The multiplex PCR for a given marker included an internal control, which allowed measurement of the integrated density of marker-specific PCR products at different cycles. The ratio of the DNA of target band over control fragment was calculated for the patient and compared to the normal control. Figure 1 shows an example of the quantitative PCR analysis which identified the presence of deleted markers with a 2:1 (Figure 1A) and a non-deleted marker with 1:1 ratio (Figure 1B) of control to patient. Such results on all markers identified the proximal and the distal breakpoints associated with the patient's deletion (Figure 2) as explained below.

#### Proximal breakpoint of 22q11 deletion

Examination of the proximal end of the 22q11 region assessed markers WI307 to HIRA. The results show that markers WI307, D22S427 and RH48663 are not deleted while markers RH48348, G18185, NIB1910, A00IT42, Cit-trans-11R, D22S609 and HIRA are deleted in this patient. These results allowed us to map the proximal breakpoint between the markers RH48663 and RH48348. Custom primers were designed at regular intervals between RH48663 and RH48348, and semi-quantitative PCR analysis situated the proximal breakpoint between primers P15565 and P15567 (Figure 2). The sequence between these two primers, designated as the proximal shortest region of the breakpoint origin (SRBO), is 1750 bp long.

#### Distal breakpoint of 22q11 deletion

Initial analysis at the distal part of the 22q11 region was conducted targeting the 9 known genetic markers between D22S944 to D22S936. Semi-quantitative PCR results placed the distal breakpoint between the genetic markers D22S1138 and SHGC-145314, located approximately 80 kb apart in the 22q11 region. Therefore, a more precise mapping of the distal breakpoint was attempted using a new set of custom primers that interrogated the 80 kb region at regular intervals. Our results determined that the distal breakpoint was located between P17731 and P177318 (Figure 2). The distance between these two markers was estimated to be 1172 bp and was designated as the distal SRBO.

### The nature of deletion

The presence of D22S936 and D22S636 in two copies in this patient indicated that the distal breakpoint lay in the distal region of the common 3 Mb TDR. Further, the mapping of deleted markers including D22S264 suggested that the size of the deletion was larger than 1.5 Mb yet less than 3 Mb since the distal breakpoint was flanked by D22S1138 and SHGC-145314. This information indicated that the hemizygous deletion in this patient was approximately 2.3 Mb. This novel deletion (approximately 17001842 – 19356929) contains ~75 genes and overlaps the 3 Mb deletion common to this genomic area. The proximal breakpoint of this deletion is localized in the LCR region common to most reported deletions, but the distal breakpoint represents a novel site. Next, we aimed to identify the sequence composition of the two SRBOs and the novel sequence of the derived chromosome. The results of sequencing of the proximal (1750 bp) and distal (1172 bp) SRBOs showed considerable sequence identity. Specifically, a 90% similar 270 bp shared sequence between the breakpoint regions (SSBR) was found to be present. The sequencing of the deletion-derived chromosome supported the presence of the shared sequence and yielded a continuous sequence that was a recombination of the proximal and distal fragments.

### Breakpoint sequence and origin analysis

#### Characterization of SSBR

Extensive blast analysis was performed using the SSBR sequence in both 22qTDR sequence and its repeat element data. When SSBR sequence was compared with the repeat-masked 22qTDR, the SSBR sequence was masked and not identified in the commonly deleted region. However, the SSBR generated a large number of hits with the non-RepeatMasked 22qTDR sequence. Such results suggest that the SSBR identified in this research belongs to a family of repeat elements and similar sequences were identified at 342 locations with >90% identity. Six of these matches were contained in the proximal SRBO, located in a region between RH48663 and RH48348 and 8 such matches were located in the distal SRBO between D22S1138 and SHGC-145314.

Further, we explored the origin of the SSBR sequence by comparing it with different repeat elements. When the SSBR was compared with different repeat sequences considering a minimum alignment length of 200, the SSBR aligned with SINE/Alu elements 91% of the time and only 7% and 2% of time with LINE/L1 (LINE-1) and LTR (long terminal repeats)/MaLR (mammalian apparent LTR-retrotransposon) sequences respectively (Table 2). The SSBR sequence did not show any similarity to other simple or medium reiteration sequence (MER) type repeats. Such results imply that this sequence has its origin in the SINE/Alu group of sequences.

**Table 2:** Alignment of SSBR sequence with different repeat elements.

Sequence length interval	SINE/Alu	LINE	Simple repeats	DNA/MER1_type	LTR/MALR
270–279	72	7	0	0	1
260–269	70	0	0	0	
250–259	32	1	0	0	1
240–249	4	1	0	0	1
230–239	10	3	0	0	
220–229	4	1	0	0	
210–219	2	0	0	0	1
200–209	4	2	0	0	1
Total	198	15	0	0	5

#### Relationship of SSBR with PDR breakpoints

**Figure 3:**
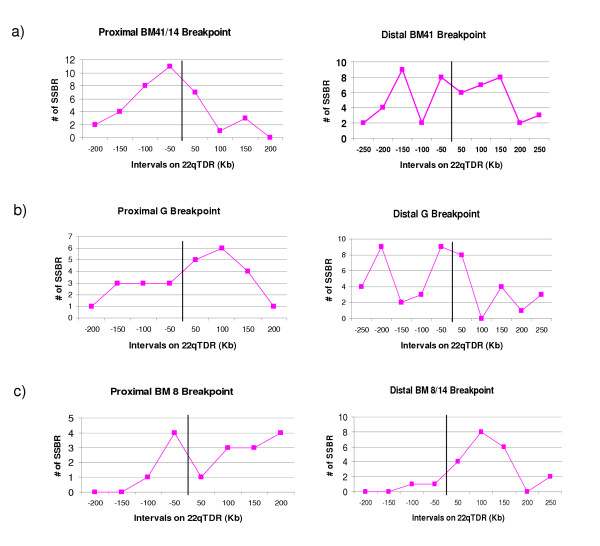
Relationship between SSBR occurrence and approximate PDR breakpoint positions on 22qTDR. The x-axis marks/represents the interval on the 22qTDR in 250-200 Kb regions on both the 5' and 3' end of the PDR breakpoint (marked in the center by a vertical line). Y-axis displays the number of SSBRs present in each 50 Kb interval on x-axis.

Integrated Genome Browser allows comparative visualization of sequence annotation side by side organized in tracks. These annotations can be viewed at the nucleotide level. Chromosome 22 Refseq annotation (*H. sapiens *May 2004 build data) [34], LCR locations, SSBR alignment to the 22qTDR, repeat elements and PDR breakpoints data were loaded as individual tracks into the IGB [see Additional file 2]. A comparative analysis was performed to identify the occurrence of SSBRs within the 22qTDR in relation to previously known breakpoints. The majority of the PDR breakpoints lie in or near the center of the SSBR cluster on 22qTDR (Figure 3). The proximal SRBO is located just 5' of the typical 3 Mb deletion and, along with the proximal BM41 (Table 1) breakpoint, is situated in the middle of a SSBR cluster (Figure 3a). For example, the SSBR nearest to the proximal BM41 is only 108 bp from the 3' breakpoint and 3 kb from the 5' breakpoint. The distal SRBO is located between the classical 1.5 and 3 Mb deletion. Interestingly, the distal BM41, proximal G and distal G (Table 1) breakpoints are harbored in the center of SSBR clusters (Figure 3a and 3b). The closest SSBR is only 294 bp 3' and 385 bp 5' to the distal BM41 breakpoint, 416 bp 3' to the distal G breakpoint and 161 bp 3' to the proximal G breakpoint. The proximal BM8 and distal BM8/14 breakpoints are located just 3' and 5' to SSBR clusters (Figure 3c). The nearest SSBR sequence is 2 kb 3' of the distal BM8/14 breakpoint and only 9 kb from the proximal BM8 breakpoint. Presence of the SSBR sequence in multiple copies near all breakpoints suggests that it may confer susceptibility to chromosomal rearrangements and help to explain the high frequency of deletions associated with the 22q11 region.

## Discussion

The 22q11 region continues to attract genetic interest by virtue of its involvement in common microdeletions (e.g. deletion in 22q11), presence of unusual repeats, and as the potential site responsible for a number of syndromes and diseases. Of particular interest is the molecular basis of breakpoints that occur with a biased distribution across the genome. In this context, a number of LCRs, a prevalent feature of this region, have been implicated in microdeletions [15-19]. It is also clear that not all LCRs are equally involved in deletions. Although most known deletions share a common proximal breakpoint, the distal breakpoints are not always the same resulting in a deletion ranging from 3.0 Mb (the most common) to 1.5 Mb in size. Recently, a number of rare deletions of intermediate size (2.8 Mb) have also been reported [35]. This report describes another such case with a deletion size of 2.3 Mb. Detailed sequencing of the breakpoints, included in this report, has allowed identification of an SSBR with potential to undergo intra-chromosomal pairing and recombination causing this novel 2.3 Mb. deletion. The significance of such SSBRs potentially located at other breakpoints is discussed below.

### Defining breakpoints in a 22q11 deletion

Molecular characterization of the deletion in this patient utilized a total of 40 markers distributed across the 22q11 region using semi-quantitative PCR and was found to be highly effective and reliable. Mapping of the deleted and non-deleted markers on the chromosomal sequence placed the proximal breakpoint site at 17001842 bp and the distal site at 19356929 bp (Table 1). This represents a novel deletion of 2.3 Mb length and is contained within the most common 3.0 Mb TDR (Figure 2) [36]. The proximal breakpoint matches with other reported deletions in the LCR region, but the distal breakpoint represents a new breakpoint site that does not correspond to known LCRs of 22q11. Interestingly, the two breakpoint regions of this patient share a short 270 bp SSBR sequence with >90% similarity. We have hypothesized that this sequence may facilitate intra-chromosomal pairing and cross-over resulting in a 2.3 Mb deletion in the gamete of one of the parents. Our SSBR sequence shares extensive sequence similarity with SINE/Alu as well as some similarity with LINE and LTR/MaLR sequence. The predicted origin of this sequence appears to involve the common repeats of the human genome and is embedded in the LCRs of the chromosome 22.

### Origin and mechanism of the 2.3 Mb 22q11 deletion

**Figure 4:**
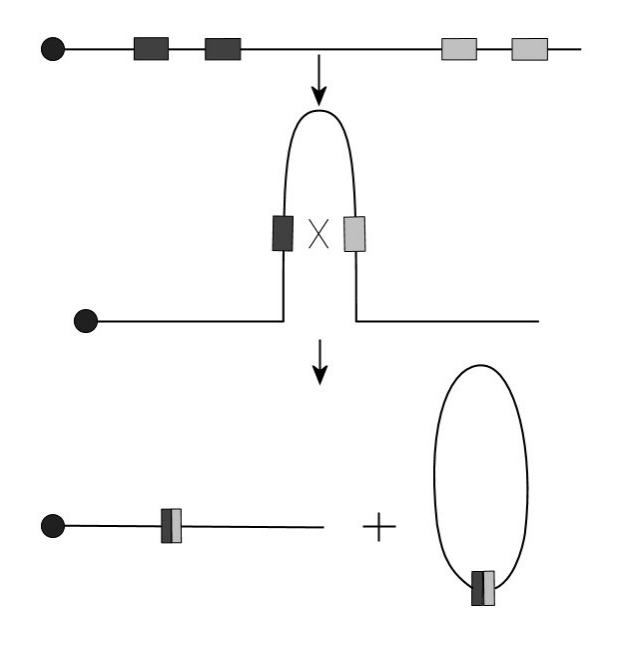
Model for intra-chromosomal rearrangement/recombination between SSBR sequences leading to deletion in chromosome 22. Filled circles indicate the centromeres. Dark and grey boxes represent different SSBR sequence blocks.

We propose that the mechanism of this novel deletion is not any different from other deletions of this region, even though its distal breakpoint was not contained in an LCR area. This may be due to the fact that the two breakpoints share enough sequence similarity to SINE/Alu elements to facilitate chromosomal rearrangement (Figure 4). This model was based on the generic homologous recombination mechanism proposed earlier [22,37]. Extensive similarity of the SSBRs to Alu elements is also backed by the fact that Alu elements are also approximately 280 bp in length [24] and different subfamilies of Alu elements have formed and become mobilized at different times in the evolution of primate species [38].

The role of SINE/Alu repeats in modulating the architecture of the human genome in association with human disorders and in mediating gene rearrangements has also been established [25,26]. The most frequent mechanisms by which Alu elements modulate architecture are transposition and unequal homologous recombination, both of which have been linked to human diseases [26]. Babcock et al., 2003, [32] have shown that Alu-mediated rearrangements are responsible for shaping the genes within LCR22s and found evidence of unequal crossover mechanisms between Alu elements that are likely responsible for the rearrangements. Homology between Alu elements may be responsible for misalignment of chromosomes during meiosis. Interestingly, the majority of human genetic disorders caused by homologous recombination between repetitive sequence are the result of Alu-Alu recombination [25,26,39]. Thus, it is highly likely that the two SRBO regions will have the potential to pair intra-chromosomally through Alu like SSBR sequence. Further, a crossover between the SRBO by SSBR may have generated this 2.3 Mb deletion in this patient.

Besides Alu, SSBR-like sequence mediated crossover mechanisms have been reported for a number of deletions in the human genome. For example, in the case of hereditary neuropathy with liability to pressure palsies (HNPP, OMIM 162500) [40], VCFS [41,42] and in ADU breakpoint [20] with a sequence size range of 550 to 750 bp. The presence of the SSBR sequence in the proximal and distal SRBOs suggests a similar rearrangement (Figure 4) in the 22qTDR, resulting in a 2.3 Mb deletion in this patient. The mechanism proposed also appears to be common for deletions in humans at a number of sites in the human genome. These include the 7q11.23 region involved in Williams syndrome [43], 17p11.2 involved in Smith-Magenis syndrome [44] and 15q11-13 involved in Prader-Willi syndrome [45], among others. Also, the presence of SSBR in multiple copies around the SRBO indicates that the SSBR sequence is particularly susceptible to genome rearrangement by homologous recombination. Identification of the SSBRs associated with most such deletions will be valuable in refining this generic model to include sequence specificity, as identified in this report.

## Conclusion

This research has identified a novel 2.3 Mb deletion in the 22q11 region. Analysis of the sequence at the breakpoints revealed a SSBR that showed strong sequence similarity to SINE/Alu elements. This Alu like SSBR sequence fragment is distributed across the 22qTDR with a higher frequency in the previously known breakpoint regions. Evidence provided above suggests that, this SSBR may be involved in the mispairing of the SRBOs or LCRs during homologous recombination resulting in chromosomal rearrangement leading to deletion in the 22q11 region.

## Competing interests

The author(s) declare that they have no competing interests.

## Authors' contributions

RKU and YZ made equal contribution to this manuscript. YZ performed the molecular genetic analysis. RKU carried out the bioinformatics analysis and manuscript preparation. Patient assessments were carried out by RO and JR. Clinical description of the patient was completed by VMS. Cytogenetic and FISH analysis were carried out by Y-SF and YZ. SMS adopted the research and provided guidance.

## Pre-publication history

The pre-publication history for this paper can be accessed here:



## Supplementary Material

Additional File 1 Supplementary table 1. List of primers used for the molecular characterization of the 22q11 deletion. Click here for file

Additional File 2 Additional Figure 1. Short description: A comparative analysis of the SSBRs, LCRs and approximate PDR positions on 22qTDR using the Integrated Genome Browser (IGB). Click here for file
