# Supplementary material for: Breakpoint Associated with a novel 2.3 Mb deletion in the VCFS region of 22q11 and the role of Alu (SINE) in recurring microdeletions
Source: BMC Med Genet. 2006 Mar 2;7:18. doi: 10.1186/1471-2350-7-18 (PMC1413517; doi:10.1186/1471-2350-7-18)
Supplement: Additional File 1 — Supplementary table 1. List of primers used for the molecular characterization of the 22q11 deletion. [file 1471-2350-7-18-S1.doc]

**Supplementary table 1.** List of 40 primers in consecutive order (centromere to telomere) used for the molecular characterization of the 22q11 deletion. Eighteen primer pairs for sequence-tag site (STS) markers (marked *) and 22 newly designed unique primer (prefixed with P) pairs were employed. Multiple primers within the same genetic marker were numbered consecutively separated by a dot (.) after the marker name.

| Primer name | Sequence of forward primer (5'-3") | Sequence of reverse primer |
| --- | --- | --- |
| *WI307.1 | GTA ATT TTC TCC TAC ATT CTT AGG C | ATT ATT TGC TCA ACA TTT AAA GAC A |
| WI307.2 | GTA ATT TTC TCC TAC ATT CTT AGG C | CAC TGTAGC ATA GAA ATT TGG CT |
| *D22S427.2 | TGC TGT TTT GTA GAG TGT TTA GAC | GAT GGA GTC TTG CTC TGT CGC C |
| D22S427.3 | ACT TTG GGA GGC CGA GGC AG | GAT GGA GTC TTG CTC TGT CGC C |
| D22S427.4 | TGC TGT TTT GTA GAG TGT TTA GAC | GTG CCC AGC CGT ATT T |
| *RH48663 | TTC TCC TTC TTC CAT CCC CT | TCT GCA TGC TCA GCA ACA C |
| P15560 | CTT GCT AAG GGA GGA GAC CA | TAA CCG ATT AGG TCA GGG G |
| P15564 | CCA ACG TGC CAC CTT TAA GAG | GAC CCT CGC AGT GAG TGT TG |
| P15565 | GGT GGT GGT CGC CTG TAG TT | GGA GTC TTG CTC TGT CAC CCA |
| P15567 | GCC AGA GGA TAG GGA GTG C | GTG GAA GCA GTC AAA CAG AAC |
| P155675 | AGG AGA AAG TTG TGG AAA GGG AC | AGA ATC GCT TGA ACC TGG GA |
| P15568 | GCC AGA GGA TAG GGA GTG CC | GGT CAC AGA GGA AGA TGT TGG C |
| *RH48348 | TCC AGG GAC ACT GAA AGT ATC C | AAT CAT GCG CGA AAG AGT TT |
| *G18185 | TTC TCA ACT CCC CCT GTC C | CGA TAG CAG TGA GGT GCA AA |
| *NIB1910 | GCA GGA ACA GTG GTT TAT TG | GAA CTC TCC TCG AAT GTG TG |
| *A001T42 | ACA GCA TCT TAA GAA AAG TTA GT | GAT TTG ATA CTG TAC ATT GTC T |
| *Cit-trans11R | GAT TCT AGA AGG GCA CTG TC | TGC GGC CTC TCT AGG CTT AG |
| *D22S609 | ATC CCA AAG TAC TTA CAA AGC A | TGG GAG AGC TTG GAG TTT AA |
| D22S609.2 | ATC CCA AAG TAC TTA CAA AGC A | GAG GTA TCT GTA GTG TCT TGA GTG |
| HIRA | TGT CAG CCT ACA CAG GCT CCT G | CTC AGT CGC AGG CGT TGT CA |
| HIRA.2 | TGT CAG CCT ACA CAG GCT CCT G | GGA TCT GTG GGT TAG CCA GCC |
| *D22S944 | CAT GTG AAA GAT GCT ACT TCC | ATC CCA TGC TCC TCC CCA T |
| *D22S931 | GTG AGA TGG ACC GGA ACT TTG | CTA CCA GGG CAA TCC TGA GC |
| *D22S264 | ATT AAC TCA TAA AGG AGC CC | CAC CCC ACC AGA GGT ATT CC |
| *D22S1138 | TCC TCT CTA TGT TGG AAA TGT CAG | ATG ATC CCA GGT GAC TCA GAT TC |
| P17690 | CCT CTC GCC TCA GCC TCC AG | CTG TTC ACC TGC TGA TGG ATG GC |
| P17723 | CGG CAT AGA GGT TCC ACT CG | CAC GGT CTT CTT CTC GCA CG |
| P17731 | GAA AGT TGA GAT TTG GAG GGA AGA | TTG CTG TTA CGA AGC CTA CGA TT |
| P177318 | AGA GTC GCA CAT CAC CAC AAT C | AAG AAG CCA GCC ACA AGA TCA |
| P177325 | AGC TGG TCT CCA ACT CCT GAG C | AAA GCC ACA TGC TCA AAG AAC AC |
| P17732 | TCA GCC TCT TGA GTA GCG GG | TGT GCT CTC ACT CAT TCC TGT CA |
| *SHGC145314 | TCC TGG ATC TTA CTA GTT TGC GG | TGA TTG GAG ATG AGT AAG CCA CA |
| *RH78571 | GCA GTC CAT GGC GTT ACC | TGA GGA GGG GAC CTT CAA G |
| *D22S1026 | GTT TCC ATT CCA ACA ACG AG | GGA GAA AGG CTT GAG TGA CA |
| *D22S936 | CAA TCT TGG CAG CCA GTT TAG | CAG CAT CTT CCT GGT GGC C |
| D22S936.2 | CAA TCT TGG CAG CCA GTT TAG | CAT CTC CTT ACC TCT CCT GAT C |
| D22S936.3 | CAA TCT TGG CAG CCA GTT TAG | GAG GTG GAG GTT GCA GTG AG |
| *D22S636 | AAC CTT CTG ATG GCT CCT CT | CAT GGA GCT GAC ACT GAG TG |
| D22S636.2 | CAT GGA GCT GAC ACT GAG TG | CTT TGT GAG CCA GAA ATC GCC TG |
| D22S636.3 | CAT GGA GCT GAC ACT GAG TG | GTC TGA GTT TCC ATC ATG GTG |
|  |  |  |
