# Supplementary material for: Breakpoint Associated with a novel 2.3 Mb deletion in the VCFS region of 22q11 and the role of Alu (SINE) in recurring microdeletions
Source: BMC Med Genet. 2006 Mar 2;7:18. doi: 10.1186/1471-2350-7-18 (PMC1413517; doi:10.1186/1471-2350-7-18)

**Additional Figure 1.** A comparative analysis of the SSBRs, LCRs and approximate PDR positions on 22qTDR using the Integrated Genome Browser (IGB). The tracks (each section on each left column from bottom to top) represent 1. Coordinates along the 22q11 region, 2. SSBR (noted as CSBRs representing each vertical line on the right pane) alignment to 22qTDR, 3. Current and previously known deletions (yellow horizontal bars), 4. LCR locations (yellow horizontal bars on the right pane corresponding to the LCRs section on the left) and 5. Chromosome 22 Refseq annotations (top most track). White arrow indicates the location of specified breakpoints from table 1.

**a)** Proximal SRBO-BM41/BM14 Distal BM41

**
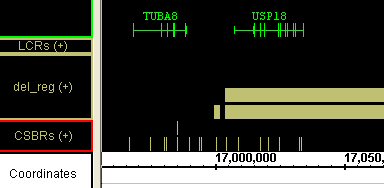
**
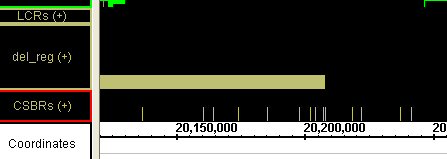


**b)** Distal SRBO

**
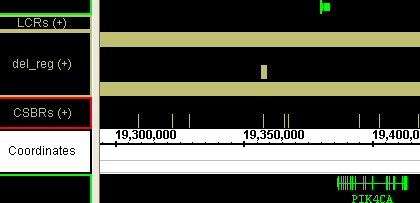
**

**c)** Proximal G Distal G


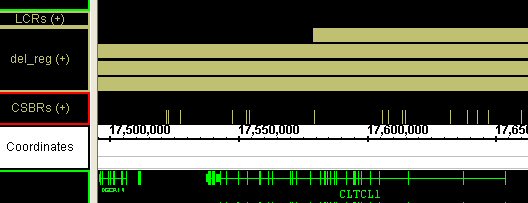

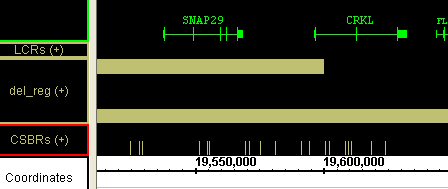


**d)** Proximal BM8 Distal BM8 and BM14


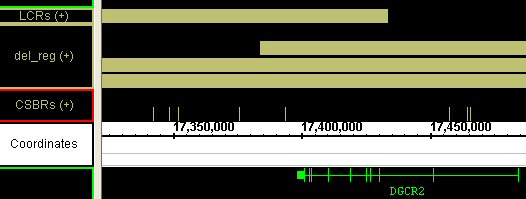

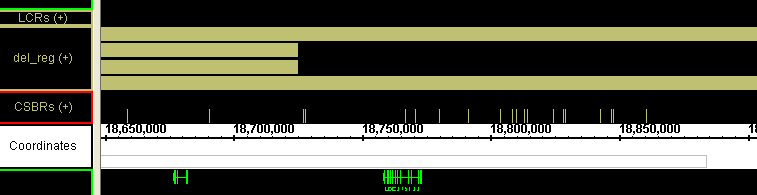

Supplement: Additional File 2 — Additional Figure 1. Short description: A comparative analysis of the SSBRs, LCRs and approximate PDR positions on 22qTDR using the Integrated Genome Browser (IGB). [file 1471-2350-7-18-S2.doc]
